# Maternal Arsenic Exposure, Arsenic Methylation Efficiency, and Birth Outcomes in the Biomarkers of Exposure to ARsenic (BEAR) Pregnancy Cohort in Mexico

**DOI:** 10.1289/ehp.1307476

**Published:** 2014-10-17

**Authors:** Jessica E. Laine, Kathryn A. Bailey, Marisela Rubio-Andrade, Andrew F. Olshan, Lisa Smeester, Zuzana Drobná, Amy H. Herring, Miroslav Stýblo, Gonzalo G. García-Vargas, Rebecca C. Fry

**Affiliations:** 1 Department of Epidemiology, and; 2 Department of Environmental Sciences and Engineering, University of North Carolina at Chapel Hill, Gillings School of Public Heath, Chapel Hill, North Carolina, USA; 3 Facultad de Medicina,Universidad Juárez del Estado de Durango, Gómez Palacio, Durango, México; 4 Carolina Population Center, University of North Carolina at Chapel Hill, Chapel Hill, North Carolina, USA; 5 Department of Nutrition, and; 6 Department of Biostatistics, University of North Carolina at Chapel Hill, Gillings School of Public Heath, Chapel Hill, North Carolina, USA

## Abstract

Background: Exposure to inorganic arsenic (iAs) from drinking water is a global public health problem, yet much remains unknown about the extent of exposure in susceptible populations.

Objectives: We aimed to establish the Biomarkers of Exposure to ARsenic (BEAR) prospective pregnancy cohort in Gómez Palacio, Mexico, to better understand the effects of iAs exposure on pregnant women and their children.

Methods: Two hundred pregnant women were recruited for this study. Concentrations of iAs in drinking water (DW-iAs) and maternal urinary concentrations of iAs and its monomethylated and dimethylated metabolites (MMAs and DMAs, respectively) were determined. Birth outcomes were analyzed for their relationship to DW-iAs and to the concentrations and proportions of maternal urinary arsenicals.

Results: DW-iAs for the study subjects ranged from < 0.5 to 236 μg As/L. More than half of the women (53%) had DW-iAs that exceeded the World Health Organization’s recommended guideline of 10 μg As/L. DW-iAs was significantly associated with the sum of the urinary arsenicals (U-tAs). Maternal urinary concentrations of MMAs were negatively associated with newborn birth weight and gestational age. Maternal urinary concentrations of iAs were associated with lower mean gestational age and newborn length.

Conclusions: Biomonitoring results demonstrate that pregnant women in Gómez Palacio are exposed to potentially harmful levels of DW-iAs. The data support a relationship between iAs metabolism in pregnant women and adverse birth outcomes. The results underscore the risks associated with iAs exposure in vulnerable populations.

Citation: Laine JE, Bailey KA, Rubio-Andrade M, Olshan AF, Smeester L, Drobná Z, Herring AH, Stýblo M, García-Vargas GG, Fry RC. 2015. Maternal arsenic exposure, arsenic methylation efficiency, and birth outcomes in the Biomarkers of Exposure to ARsenic (BEAR) pregnancy cohort in Mexico. Environ Health Perspect 123:186–192; http://dx.doi.org/10.1289/ehp.1307476

## Introduction

The World Health Organization (WHO) has established guidelines suggesting that levels of arsenic (As) in drinking water should not exceed 10 μg/L (WHO 1993). Several countries including the United States, India, Taiwan, Vietnam, and Japan have adopted the WHO’s guidelines in the establishment of their maximum contaminant levels (MCL) for As in drinking water, yet others have a higher MCL including Mexico at 25 μg As/L and Bangladesh at 50 μg As/L (Chen and Chiou 2011; Secretaría de Salud–Estados Unidos Mexicanos 2000; Nordstrom 2002). Even with these guidelines in place, there are currently millions of people worldwide who are drinking water with levels of As that greatly exceed the WHO standard or national standards (Centeno et al. 2007; Uddin and Huda 2011).

Exposure to elevated levels of inorganic arsenic (iAs) in drinking water is a major public health concern because iAs has been linked with numerous adverse health outcomes (Kapaj et al. 2006; Rahman M et al. 2009). The detrimental effects from long-term exposure include skin lesions, cardiovascular disease, peripheral vascular disease, diabetes mellitus, and cancers of the urinary bladder, skin, lung, liver, and prostate (Del Razo et al. 2011; International Agency for Research on Cancer 2004; Maull et al. 2012; Rahman M et al. 2009; Tseng 2007b). It is also evident that certain populations, including pregnant women and their unborn children, are particularly susceptible to the adverse effects of iAs exposure (Vahter 2009). For instance, prenatal iAs exposure has been associated with a greater risk of adverse pregnancy outcomes such as preterm birth, low birth weight, and/or fetal loss (reviewed by Vahter 2009). In addition, exposure to high levels of iAs *in utero* or in early childhood has been associated with multiple adverse health outcomes, including both cancer and noncancer end points, later in life (Smith et al. 2006). The biological mechanisms that determine the nature and severity of adverse effects in iAs-exposed populations involve multiple factors such as the dose and duration of iAs exposure, efficiency of iAs metabolism, genetic background, nutritional status, and coexposure to other toxicants (Gebel 2000; Kapaj et al. 2006; Vahter and Concha 2001).

Along with life stage at time of exposure, the efficiency of iAs metabolism is a well-documented risk factor for the development of several iAs-associated diseases (reviewed by Tseng 2007a). In humans, iAs has a biological half-life of approximately 24 hr (Buchet et al. 1981) and is metabolized to produce monomethylated and dimethylated arsenicals (MMAs and DMAs, respectively). Six major arsenicals associated with iAs exposure and metabolism have been detected in human urine, namely arsenite (iAs^III^), arsenate (iAs^V^), monomethylarsonous acid (MMA^III^), monomethylarsonic acid (MMA^V^), dimethylarsinous acid (DMA^III^), and dimethylarsinic acid (DMA^V^) (Thomas et al. 2001; Vahter 2002). Generally, total urinary arsenic in iAs-exposed individuals is composed of 10–20% total (trivalent + pentavalent) iAs, 10–20% total MMAs, and 60–80% total DMAs (Vahter 1999, 2002). In contrast to iAs, organic arsenicals such as arsenobetaine, arsenocholine, and arsenosugars are derived from food sources, primarily seafood (Choi et al. 2010). Because arsenosugars can be metabolized to DMAs, the presence of DMAs in urine may reflect exposure to both iAs and organic arsenic compounds (Choi et al. 2010). High urinary proportions of MMAs and high ratios of MMAs/DMAs, which are thought to be indicators of an inefficient methylation of iAs, have been associated with the development of several adverse outcomes in humans including urinary bladder cancer, non-melanoma skin cancers, carotid atherosclerosis, and chromosomal aberrations (reviewed by Tseng 2007a). It is known that pregnancy can alter the metabolism efficiency of iAs (Gardner et al. 2012; Hopenhayn et al. 2003b). However, the impact of this altered metabolism on pregnant women’s or children’s health is not well established.

We set out to understand the effects of iAs exposure and iAs metabolism during pregnancy on maternal/fetal health, with the ultimate aim of increasing awareness and reducing exposures to iAs. To this end, we established the Biomarkers of Exposure to ARsenic (BEAR) prospective pregnancy cohort. Women recruited to participate in the BEAR project reside in the Gómez Palacio area, which is located in the state of Durango in the Lagunera region of Northern Mexico. It is believed that > 450,000 people are exposed to levels of iAs in drinking water that exceed 50 μg/L in Mexico, including residents of Lagunera and Durango (Bundschuh et al. 2012). Multiple adverse health outcomes have been associated with high iAs exposure in Lagunera, including skin lesions (Del Razo et al. 1997) and diabetes mellitus (Del Razo et al. 2011). The effects of iAs exposure on pregnant women and their newborn children have not been studied in this region. In this study we investigated the association between iAs exposure during pregnancy, iAs metabolism efficiency, and birth outcomes in Gómez Palacio, Mexico.

## Methods

*Study design*. BEAR participants, adult women, were recruited during the time frame of August 2011 through March 2012 at the General Hospital of Gómez Palacio. Recruitment took place before delivery, usually within 24 hr of birth. The mean gestational age at birth was 39 weeks (range, 34–42 weeks). All procedures associated with this study were approved by the institutional review boards of Universidad Juarez del Estado de Durango (UJED), Gómez Palacio, Durango, Mexico, and the University of North Carolina at Chapel Hill (UNC-Chapel Hill). For each woman participation requirements at the time of recruitment included *a*) 1 year minimum residence in the Gómez Palacio region, which included urban locations of Gómez Palacio and surrounding rural locations, *b*) confirmation of a pregnancy without complications such as eclampsia or preeclampsia, and *c*) good overall health status (i.e., no signs of chronic or acute disease). A total of 221 women were approached for the study. Of those, 93% (*n* = 206) provided informed consent for participation in the study. Six women were not included in the study as a result of confirmation of a twin pregnancy (*n* = 1; 0.5%) or sample collection failure (*n* = 5; 2.4%). A social worker administered questionnaires to the study participants to collect the following information: age, education, occupation, time living at residence, smoking status and alcoholic beverage consumption during pregnancy (both defined as yes or no and frequency), daily prenatal supplement intake (yes or no), residence location (urban or rural), seafood consumption (yes or no), source and daily consumption of drinking and cooking water, and source of bathing water. In addition, information on previous pregnancy outcomes including number of pregnancies and number of previous pregnancy losses was gathered from questionnaires. Information on birth outcomes/measures including newborn birth weight, newborn length, gestational age, head circumference, placental weight, and 5-min Appearance, Pulse, Grimace, Activity, Respiration (APGAR) score was gathered at time of delivery by the physician (Montgomery 2000). Data on adverse outcomes were collected, including preterm birth (gestational age < 37 weeks), low birth weight (LBW; < 2,500 g), small for gestational age (SGA; birth weight < 10th percentile), and large for gestational age (LGA; birth weight > 90th percentile). SGA and LGA categories were based on newborn data collected from northern regions of Mexico (Montes-Núñez et al. 2011; Rios et al. 2008).

*Sample collection*. Within 4 weeks of newborn delivery, a drinking-water sample was collected by the research team at the homes of each of the study participants. Drinking-water samples were collected based on the subjects’ primary drinking-water source (i.e., tap or bottled water). Maternal spot urine samples were collected at the hospital before birth, immediately transferred to cryovials, and placed in liquid nitrogen. Aliquots of urine samples were shipped on dry ice to UNC-Chapel Hill and immediately stored at –80°C. It was not possible to inform the women of the levels of iAs in their drinking water during their pregnancies because these results were not available before the birth of their children. However, the women were informed within 3 months of delivery.

*Detection of arsenic in drinking water and urine*. The concentrations of iAs in drinking water (micrograms As/L; DW-iAs) were measured at UJED, Mexico, using hydride generation–atomic absorption spectrometry (HG-AAS) supported by a FIAS-100 flow injection accessory system as described previously (Devesa et al. 2004; Le and Ma 1998). The Trace Elements in Water standard reference material (SRM 1643e) (National Institute of Standards and Technology, Gaithersburg, MD) was used for quality control. The limit of detection (LOD) for iAs in drinking water by HG-AAS was 0.46 μg As/L.

All urine analyses were conducted at UNC-Chapel Hill. Because of the capacity of trivalent arsenicals to oxidize to pentavalent forms in urine, the accurate measurements of urinary iAs^III^, MMA^III^, and DMA^III^ were not possible after transport to UNC-Chapel Hill (Valenzuela et al. 2005). Concentrations of U-iAs, U-MMAs, and U-DMAs were determined by HG-AAS with cryotrapping (Devesa et al. 2004; Hernández-Zavala et al. 2008, 2009). Five-point calibration curves were prepared using pentavalent iAs, MMAs, DMAs standards (> 98% pure) as described previously (Hernández-Zavala et al. 2008), and the SRM 2669 Arsenic Species in Frozen Human Urine (National Institute of Standards and Technology) was used for quality control (Del Razo et al. 2011). The LODs for U-iAs, U-MMAs, and U-DMAs were 0.2, 0.1, and 0.1 μg As/L, respectively.

The specific gravity (SG) of each urine sample was measured using a handheld refractometer (Reichert TX 400 #13740000; Reichert Inc., Depew, NY). To account for differences in water intake/differential hydration, concentrations of U-iAs, U-MMAs, and U-DMAs in each urine sample were adjusted using the following equation: iAs × (mean SG – 1)/(individual SG – 1) as previously described (Nermell et al. 2008; Yassine et al. 2012). Urinary concentrations of U-tAs (sum of iAs, MMAs, DMAs) were reported as SG-adjusted values (micrograms As/L urine).

*Statistical analysis*. Data were analyzed using the statistical package SAS 9.3 (SAS Institute Inc., Cary, NC). Arsenical concentrations in drinking water and urine that were below their respective LODs were assigned a value equal to the LOD divided by the square root of 2. To determine the efficiency of iAs metabolism, the proportion of total urinary As comprising the individual arsenicals iAs, MMAs, and DMAs (e.g., metabolism indicators) was calculated for each study subject. A Spearman rank correlation was calculated to quantify the relationship between DW-iAs and U-tAs.

Differences in DW-iAs were analyzed based on maternal characteristics (i.e., reported drinking- and cooking-water source as tap or bottle, reported seafood consumption, location of residence, and maternal smoking status and alcohol consumption). The nonparametric Wilcoxon rank sum test was used because of the failure of DW-iAs to achieve normality in transformations. Differences in log-transformed U-tAs were analyzed based on maternal characteristics using Student’s two-sample *t*-test. Age- and education-based comparisons of levels of DW-iAs and U-tAS were analyzed using a Kruskal–Wallis test and analysis of variance, respectively.

We used multivariable adjusted regression models to assess the relationships between exposure or metabolism indicators (modeled as nontransformed, urinary SG–adjusted, continuous variables) and birth outcomes (modeled as continuous variables). Regression assumptions of linearity and the homogeneity of residuals were evaluated by examination of appropriate residual plots. Covariates were selected if associated with both exposure and outcome or based on their *a priori* status as known confounders using a directed acyclic graph (DAG). Additionally, if covariates changed the estimate by at least 10%, they were included in the model. Model 1 included the following covariates: maternal age at delivery (as a continuous variable), education level as a measure for socioeconomic status (SES) (< high school, high school, college, > college), smoking status (dichotomized as yes/no), and alcoholic beverage consumption during pregnancy (dichotomized as yes/no). Gestational age was not included in model 1 because of its potential role as a mediator of birth weight that can result in biased estimates (Wilcox et al. 2011). Model 2 included gestational age as a covariate for comparative purposes. Model 3 was run as part of a sensitivity analysis examining the effects of seafood consumption as a variation of model 1 that excluded seafood consumers. Regression results are presented as a change in birth outcomes/measures with a 1-unit change in exposure indicators or metabolism indicators. The relationships between covariates, exposure indicators, metabolism indicators, and sex of newborn were assessed using a Wilcoxon rank sum test. Statistical significance for all tests was set at an alpha of < 0.05.

## Results

*Maternal and newborn characteristics*. All maternal and birth characteristics are presented in Table 1. Women in this cohort were relatively healthy and free of disease with no major adverse birth outcomes observed. All pregnancies resulted in a singleton live birth. The ages of the participants ranged from 18 to 41 years (mean, 24 years), and all were of Hispanic ethnicity. Of the study cohort, approximately 75% had at least a high school education, and all had lived in their current residence for at least 1 year (mean, 20 years). Most women (97%) reported taking prenatal vitamins daily, and most women (78%) did not consume seafood during their pregnancy. For 35% of the women, the index pregnancy was their first pregnancy. A previous pregnancy loss was reported by 12% of all women. Among all pregnancies, 59% were delivered by vaginal delivery and 41% were delivered by cesarean section, a rate common in Mexico (Boyle and Reddy 2012).

**Table 1:** Selected demographic characteristics and levels of iAs in drinking water and urinary arsenicals of the participants of the BEAR study.

Characteristic	*n*^*a*^ (%) or mean, median [range]
Maternal age at delivery (years)	24, 23 [18–41]
Race
Hispanic	199 (99.5)
Education
< High school	50 (25.1)
High school	95 (47.7)
College	41 (20.6)
Post-college	13 (6.5)
Time living at residence (years)	20, 21 [1–41]
Smoking status
Nonsmokers	186 (93.0)
Current smokers	13 (7.0)
Alcohol consumption
None	159 (79.5)
Some	41 (20.5)
Prenatal vitamin daily intake	192 (97.0)
Seafood consumption
None	155 (78.3)
Some	43 (21.7)
Previous pregnancies
0	70 (35.0)
1	50 (25.0)
≥ 2	80 (40.0)
Previous pregnancy loss
0	176 (88.0)
1	18 (9.0)
≥ 2	6 (3.0)
Method of delivery
Vaginal	118 (59.0)
Cesarean section	82 (41.0)
Gestational age (weeks)
All	39, 40 [34–42]
< 37	3 (1.5)
≥ 37	197 (98.5)
Newborn sex
Male	104 (52.0)
Female	96 (48.0)
Birth weight (g)
All	3339, 3355 [1800–5120]
Male	3453, 3490 [2100–5120]*
Female	3215, 3150 [1800–4200]
LBW	4 (2.0)
SGA	28 (14.0)
LGA	19 (9.5)
Placental weight (g)	648, 640 [390–1070]
Newborn length (cm)	50, 50 [40–59]
Head circumference (cm)	35, 35 [31–38]
APGAR score	9, 9 [8–10]
Exposure measures
DW-iAs (μg As/L)	24.6, 13.0 [< LOD^*b*^–236.0]
Urinary arsenicals^*c*^
U-tAs (μg/L)	37.5, 23.3 [4.3–319.7]
U-iAs (μg/L)	2.1, 1.3 [0.14–23.0]
U-MMAs (μg/L)	2.3, 1.4 [0.12–18.2]
U-DMAs (μg/L)	33.1, 20.6 [1.4–292.5]
iAs (%)	6.1, 5.3 [0.77–45.1]
MMAs (%)	6.4, 6.0 [0.68–24.9]
DMAs (%)	87.6, 88.5 [32.7–96.7]
MMAs/iAs	1.2, 1.2 [0.13–5.5]
MMAs/DMAs	0.077, 0.069 [0.0072–0.68]
DMAs/MMAs	17.6, 14.6 [1.5–140.0]
MMAs + DMA/iAs	19.6, 18.1 [1.2–129.9]
^***a***^Differences in *n* based on missing demographic data. ^***b***^LOD for DW-iAs = 0.456 μg As/L. ^***c***^All urinary values were adjusted by SG. *Significant difference in means (*p* = 0.0003) between males and females.	

The average gestational age of the newborns was 39 weeks. Of all births, 1.5% were preterm (< 37 weeks). Of the newborns, 104 (52%) were male and 96 (48%) were female. The average weight of the newborns was 3,339 g (SD ± 500 g); males had a significantly (*p* = 0.0003) higher birth weight (3,453 g) relative to females (3,215 g). Among newborns, 2% were LBW, 14% were SGA, and ~ 10% were LGA. The average placental weight was 648 g. The newborns had an average crown to heel length of 50 cm, an average head circumference of 35 cm, and an average APGAR score of 9 (Table 1).

**Figure 1:**
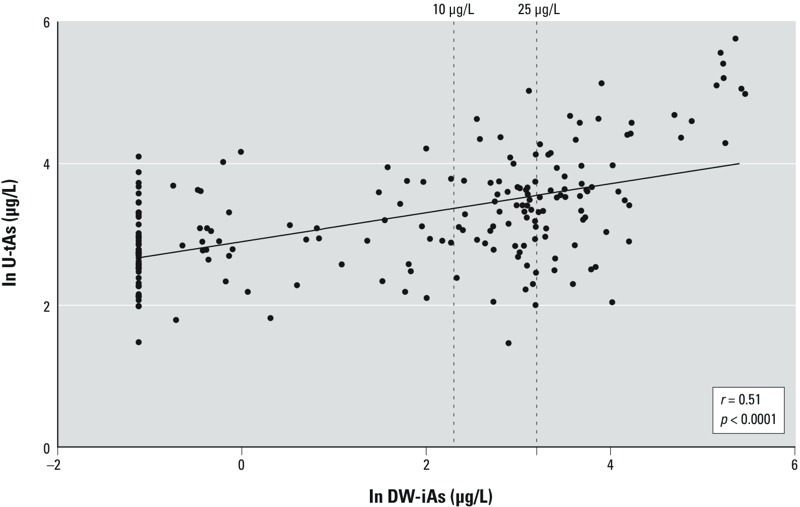
Relationship between DW-iAs and U-tAs (SG-adjusted) across the BEAR cohort (*r *= 0.51, *p *< 0.0001).

*Levels of iAs in drinking-water and urinary samples*. Levels of iAs in study subjects’ drinking water ranged from below the LOD (0.46 μg As/L) to 236.0 μg As/L (mean, 24.6 μg As/L) (Table 1). Of the 200 women, 76% had detectable levels of iAs in drinking water. In total, 107 drinking-water samples (53%) exceeded the WHO standard of 10 μg As/L, and 56 (28%) were above Mexico’s MCL of 25 μg As/L (Figure 1).

Arsenic was detected in the urine of all women. Specifically, of the 200 women, DMAs were detected in all women’s urine, and 95% of the women had levels of iAs and MMAs that were above the LOD for these arsenicals (0.2 μg As/L and 0.1 μg As/L, respectively). Across the entire cohort, there was a significant positive relationship between U-tAs and DW-iAs (*r* = 0.51, *p* < 0.0001) (Figure 1). The unadjusted sum of the urinary arsenicals (U-tAs) ranged from 1.9 to 448.2 μg/L (mean, 35.5 μg As/L) (data not shown). To control for differential hydration, SG-adjusted concentrations of U-tAs were determined and ranged from 4.3 to 319.7 μg As/L (mean, 37.5 μg As/L) (Table 1). The mean urinary concentrations of individual SG-adjusted arsenicals were 2.1, 2.3, and 33.1 μg As/L for iAs, MMAs, and DMAs respectively (Table 1). This corresponds to the proportions of U-tAs comprising 6.1%, 6.4%, and 87.6% for U-iAs, U-MMAs, and U-DMAs respectively (Table 1).

DW-iAs and U-tAs levels were analyzed in relation to demographic characteristics. Most women (55%) reported that their primary drinking- and cooking-water source was from the tap (see Supplemental Material, Table S1). DW-iAs was higher (*p* < 0.0001) for those women who reportedly drank and cooked with municipally supplied water (i.e., tap) (medians of 23.3 and 18.1 μg As/L, respectively) than for those who reportedly drank and cooked with bottled water (medians, 0.40 and 0.65 μg As/L, respectively) (see Supplemental Material, Table S1). Similarly, U-tAs levels were higher in women who reportedly drank and cooked with municipally supplied water (means, 49.5 and 42.9 μg As/L, respectively) than in those who drank and cooked with bottled water (means, 22.9 and 18.5 μg As/L, respectively) (see Supplemental Material, Table S1). There were no statistically significant differences in U-tAs (see Supplemental Material, Table S1), U-iAs, or U-MMAs (data not shown) between the women who reported consuming seafood versus those who did not; however, women who consumed seafood had higher mean levels of U-DMAs than those who did not (42.5 μg As/L vs. 30.6 μg As/L, respectively). There was no statistical difference in U-tAs associated with a woman’s age; however, women with less than a high school education had significantly higher (*p* = 0.0173) U-tAs (mean, 47.70 g As/L) than women with a high school education (mean, 37.2 μg As/L), college (mean, 32.1 μg As/L), and greater than college education (mean, 19.4 μg As/L). There was no statistical difference in U-tAs based on residence status (e.g., urban or rural environment), smoking status, nor alcohol consumption (data not shown).

*Relationship of DW-iAs and urinary arsenicals with birth outcomes*. We used multivariable regression models to examine the relationships between nontransformed DW-iAs, SG-adjusted urinary levels, and proportions of iAs, MMAs, and DMAs and birth outcomes. The primary model (model 1) adjusted for maternal age, education, smoking, and alcohol consumption, where all measured relationships represent analysis for a 1-unit change in exposure measures in association with birth outcomes/measures. DW-iAs, U-tAs, U-DMAs, and percent iAs (%iAs) were not significantly associated with any of the birth outcomes (Table 2; see also Supplemental Material, Figure S1).

**Table 2:** Multivariable regression analyses*^a^* between a 1-unit increase in exposure and metabolism indicators (DW-iAs, SG-adjusted U-tAs, U-iAs, U-MMAs, U-DMAs, %iAs, %MMAs, and %DMAs) and birth outcomes.

Variable	Gestational age (weeks)	Birth weight (g)	Newborn length (cm)	Head circumference (cm)	Placental weight (g)	APGAR score
DW-iAs
β (CI)	–0.0025 (–0.0065, 0.0061)	–0.1 (–1.7, 1.4)	–0.0051 (–0.014, 0.0038)	–0.0026 (–0.0077, 0.0026)	0.12 (–0.32, 0.57)	0 (–0.0005, 0.0005)
*p*-Value	0.22	0.89	0.26	0.33	0.58	0.98
U-tAs
β (CI)	–0.0036 (–0.0075, 0.003)	–0.58 (–2.1, 0.93)	–0.0055 (–0.014, 0.0033)	–0.0022 (–0.0074, 0.0029)	0.31 (–0.14, 0.76)	0 (–0.0004, 0.0005)
*p*-Value	0.07	0.44	0.22	0.39	0.17	0.85
U-iAs
β (CI)	–0.069 (–0.13, –0.0043)	–21.7 (–46.8, 3.4)	–0.16 (–0.31, –0.019)	–0.061 (–0.15, 0.030)	0.73 (–7.1, 8.5)	–0.0013 (–0.0097,0.0072)
*p*-Value	0.03*	0.09	0.02*	0.18	0.85	0.76
U‑MMAs
β (CI)	–0.067 (–0.13, –0.0092)	–24.4 (–46.8, –2.0)	–0.11 (–0.24, 0.018)	–0.067 (–0.14, 0.011)	–1.3 (–8.0, 5.5)	0.0004 (–0.0069, 0.0070)
*p*-Value	0.02*	0.03*	0.08	0.09	0.71	0.91
U-DMAs
β (CI)	–0.0037 (–0.0081, 0.0006)	–0.49 (–2.2, 1.2)	–0.0054 (–0.015, 0.0043)	–0.0021 (–0.0079, 0.0036)	0.38 (–0.11, 0.88)	0.0001 (–0.0005, 0.0006)
*p*-Value	0.09	0.56	0.27	0.46	0.12	0.82
%iAs
β (CI)	–0.0006 (–0.043, 0.042)	–6.4 (–22.9, 10.0)	–0.054 (–0.15, 0.041)	0.01 (–0.044, 0.065)	–3.2 (–7.9, 1.6)	–0.0002 (–0.0054, 0.0049)
*p*-Value	0.97	0.44	0.26	0.71	0.18	0.92
%MMAs
β (CI)	–0.043 (–0.098, 0.012)	–24.5 (–45.6, –3.4)	–0.082 (–0.21, 0.041)	–0.03 (–0.10, 0.041)	–9.8 (–15.8, –3.8)	0.011 (–0.0056, 0.078)
*p*-Value	0.12	0.02*	0.19	0.41	0.001*	0.73
%DMAs
β (CI)	0.011 (–0.017, 0.38)	8.9 (–1.5, 19.3)	0.041 (–0.020, 0.10)	0.0025 (–0.032, 0.037)	3.5 (0.53, 6.5)	–0.0001 (–0.0034, 0.003)
*p*-Value	0.44	0.09	0.17	0.88	0.02*	0.93
^***a***^Model 1 was adjusted for the following covariates: maternal age, education, smoking, and alcohol consumption. *Association between birth outcomes and arsenic exposure indicators, *p* < 0.05.						

There was a significant negative relationship between U-iAs and mean gestational age, with an estimated decrease of 0.069 weeks in gestational age for every 1-unit change in U-iAs [95% confidence interval (CI): –0.13, –0.0043, *p* = 0.03]. Similarly, there was an estimated decrease in mean newborn length of 0.16 cm in relationship to U-iAs (95% CI: –0.31, –0.019, *p* = 0.02). There was no significant association between U-iAs and birth weight, head circumference, placental weight, and APGAR score.

There was a significant negative association between U-MMAs and both mean gestational age (weeks) (β = –0.067; 95% CI: –0.13, –0.0092, *p* = 0.02) and mean birth weight (grams) (β = –24.4; 95% CI: –46.8, –2.0, *p* = 0.03). There were no significant associations between U-MMAs and mean head circumference, placental weight, and APGAR score. There was a significant negative relationship between %MMAs and mean birth weight, with an estimated 24-g decrease in birth weight for every unit increase in %MMAs (95% CI: –45.6, –3.4, *p* = 0.02). There was no significant association with %MMAs and mean head circumference or APGAR score. Placental weight (grams) was also negatively associated with %MMAs (β = –9.8; 95% CI: –15.8, –3.8, *p* = 0.001) and positively associated with %DMAs (β = 3.5; 95% CI: 0.53, 6.5, *p* = 0.021).

Gestational age was included in model 2 for comparative purposes (see Supplemental Material, Table S2). The inclusion of gestational age did influence the estimates, in general reducing the magnitude of the relationships observed in model 1. It is important to consider, however, the potential role of gestational age as a mediator of birth outcomes. A sensitivity analysis that excluded seafood consumers (*n* = 43, 22% of all subjects) (model 3) showed consistent directions and significance of associations between exposure and metabolism indicators and birth outcomes, as observed with model 1 (see Supplemental Material, Table S2). Maternal urinary means of iAs and urinary arsenical proportions were not statistically different (*p* < 0.05) based on the sex of the infant (data not shown).

## Discussion

The BEAR prospective pregnancy cohort in Mexico was established with a long-term goal of examining the effects of environmental exposure to iAs on the health of women and children and providing information to protect individuals from preventable exposure. The results of the present study demonstrate that pregnant women in the BEAR cohort are exposed to potentially harmful levels of iAs in their drinking water, ranging up to 236 μg As/L. More than half of the samples exceeded the WHO-established safe drinking-water guideline of 10 μg As/L. Elevated levels of arsenic have been observed in the drinking water of other exposed populations in Mexico. For instance, previously reported DW-iAs from central-eastern regions of Mexico ranged up to 1,504 μg As/L (Valenzuela et al. 2005) and up to 215 μg/L (Del Razo et al. 2011) in Hidalgo and Zimapán, respectively.

The biomonitoring data further confirmed that pregnant women in the cohort are exposed to iAs, with SG-adjusted U-tAs ranging from 4.3 to 319.7 μg As/L. Levels of U-tAs were positively correlated with DW-iAs, consistent with drinking water being a source of iAs exposure in the BEAR cohort. The level of correlation between DW-iAs and U-tAs is similar to that of other iAs-exposed populations in Mexico (Del Razo et al. 2011). The levels of U-tAs in the BEAR cohort are generally lower than levels observed in pregnant women in Bangladesh (SG-adjusted medians, 23.3 μg As/L vs. 80 μg As/L) (Vahter et al. 2006). However, the levels of U-tAs in this cohort are higher than levels observed in U.S. women (SG-adjusted mean, 37.5 μg As/L vs. creatinine adjusted geometric mean, 7.3 μg As/L) (Caldwell et al. 2009). The drinking-water samples from the present study were collected postpartum and represent a single exposure measure, so there is the potential that exposure varied over time. However, it has been demonstrated in prior research that iAs levels in drinking water can vary little temporally (Slotnick et al. 2006; Steinmaus et al. 2005). Additionally, the women in the study had lived in their current residences for at least 1 year, suggesting minimal variability in the source of iAs in drinking water and potentially for iAs exposure as well. Taken together, the data from the present study highlight a new area of concern for elevated levels of iAs in drinking water in Mexico.

When analyzed for relationships to birth outcomes, overall DW-iAs and the sum of the urinary arsenicals (U-tAs) showed no significant associations. Interestingly, there were significant relationships between levels and proportions of iAs, MMAs, and DMAs in maternal urine and selected birth outcomes. Specifically increases in U-iAs were associated with a lower means in gestational age and newborn length. Increases in U-MMAs were associated with a lower mean gestational age and birth weight. Similarly, increases in %MMAs were negatively associated with both lower mean placental weight and birth weight, with an estimated approximately 24-g decrease per unit increase in %MMAs. As total amounts of urinary arsenic as well as DMAs can be attributed in part to seafood consumption (Navas-Acien et al. 2011), a sensitivity analysis was performed on non-seafood consumers. The point estimates between U-MMAs and %MMAs with birth weight were similar between seafood and non-seafood consumers, suggesting that these associations are not affected by seafood consumption. Although it is currently unknown whether the later-life health of the infants in this study will be influenced by the observed differences in birth weight, gestational age, and newborn length, these relationships will be important given global worldwide exposure to arsenic. Thus, follow-up of these children is warranted as infants born with decreased gestational age, and changes to birth size are at risk for later life health effects.

Our data highlight the important finding that U-tAs alone was not significantly associated with birth outcomes, suggesting that the metabolism of iAs by a pregnant woman may be a better predictor of impact on both birth and placental weight, and newborn length than exposure level alone. Of note, the mean proportions of total urinary arsenicals from pregnant women in Gómez Palacio were approximately 6% for iAs, 6% for MMAs, and 88% for DMAs. These data indicate increased iAs methylation capacity (i.e., a shift to higher proportions of DMAs) relative to the proportions generally observed in exposed populations around the world, with observed ranges of approximately 10–20% for iAs, 10–20% for MMAs, and 60–80% for DMAs (Vahter 2002). U-tAs and the individual urinary metabolites were measured once during pregnancy; nevertheless, all samples represented urinary collection during the end of pregnancy—immediately before delivery. Therefore, shifts in iAs metabolism that occur differentially between trimesters of pregnancy (Hopenhayn et al. 2003a) should not influence these findings. The results in the present study highlight a relative increase in proportions of DMAs, consistent with other studies where later stages of pregnancy were associated with proportions of DMAs exceeding 80% (Concha et al. 1998; Gardner et al. 2011, 2012; Hopenhayn et al. 2003b). It has been suggested that higher DMAs:MMAs ratios indicate an increased iAs methylation capacity and may have a protective effect for both mother and fetus, because it could reflect reduced levels of the highly toxic trivalent MMAs^III^ (Vahter 2002, 2009).

An inverse relationship between DW-iAs and birth weight has been observed previously (Hopenhayn et al. 2003a; Yang et al. 2003) as well as U-tAs and size at birth (Rahman A et al. 2009). However, some studies have found no association between prenatal iAs exposure and size at birth (Kwok et al. 2006; Myers et al. 2010). These differences may be attributed to variation in study design and methodology (e.g., bias, misclassification, and/or random error), as well as inherent differences in population characteristics (e.g., exposure/consumption of iAs contaminated drinking water, genetic polymorphisms, and nutritional factors). Furthermore, studies that do not address interindividual differences in metabolism to iAs would not capture the associations observed in the present study. To our knowledge, the results from the BEAR cohort are the first to demonstrate that levels and proportions of MMAs and levels of U-iAs in maternal urine are associated with poorer birth outcomes. These findings support a growing body of literature detailing that urinary proportions of MMAs are positively correlated with detrimental health effects of iAs (reviewed by Tseng 2007a).

Although the data in the present study suggest an association between maternal methylation of iAs and infant birth weight, gestational age, and newborn length, this study is not without limitations. In addition to iAs exposure and metabolism, other factors that may influence birth outcomes include coexposure to other contaminants, gestational age, and maternal genotype. Additionally, residual confounding may remain based on maternal SES and potentially dietary sources of iAs. Together these complex factors may influence the relationships between iAs exposure during pregnancy and newborn health outcomes, and should be examined in larger cohort studies. It will be important in future research to examine the links between early-life exposure to iAs and postnatal development in the children through prospective analysis. Taken together, these data will enable a better understanding of the etiology of poorer birth outcomes associated with iAs exposure in early life.

## Conclusions

This study suggests three major findings. First, more than half of the drinking-water samples tested in Gómez Palacio exceeded the WHO’s guidelines for acceptable limits of iAs in drinking water, indicating that women drinking municipal water are at risk for elevated exposure to iAs. Second, biomonitoring data confirmed that many pregnant women had elevated levels of iAs in their urine. Third, individual differences in iAs metabolism in pregnant women were associated with birth outcomes. Specifically, elevated levels and proportions of MMAs in maternal urine were negatively associated with birth weight and gestational age, and urinary iAs was negatively associated with gestational age and newborn length. Given that these exposures to elevated iAs are occurring during pregnancy, there may also be concerns about later-life health effects for the children of the cohort. The results of this study suggest that a follow-up examination of the impacts of iAs exposure on the health of the women and their children is warranted.

## Supplemental Material

(1.6 MB) PDF
