# Supplementary material for: Maternal Arsenic Exposure, Arsenic Methylation Efficiency, and Birth Outcomes in the Biomarkers of Exposure to ARsenic (BEAR) Pregnancy Cohort in Mexico
Source: Environ Health Perspect. 2014 Oct 17;123(2):186–92. doi: 10.1289/ehp.1307476 (PMC4314242; doi:10.1289/ehp.1307476)
Supplement: (1.6 MB) PDF [file ehp.1307476.s001.508.pdf]

Supplemental Material

Maternal Arsenic Exposure, Arsenic Methylation Efficiency, and Birth Outcomes in the Biomarkers of Exposure to ARsenic (BEAR) Pregnancy Cohort in Mexico

Jessica E. Laine, Kathryn A. Bailey, Marisela Rubio-Andrade, Andrew F. Olshan, Lisa Smeester, Zuzana Drobná, Amy H. Herring, Miroslav Stýblo, Gonzalo G. García-Vargas, and Rebecca C. Fry

Table S1. Comparisons of DW-iAs and SG-Adjusted U-tAs based on selected self-reported maternal demographic characteristics.

Characteristic	*n*[a] (%)	DW-iAs (µg/L) Mean, median (range)	U-tAs (µg/L) Mean, median (range)
**Drinking water source**			
Tap	110 (55%)	40.0, 23.3 (0.326-236.0)*	49.5, 34.0 (4.3-319.7)*
No. glasses day		4.4, 4 (1-12)	
Bottled water	90 (45%)	5.8, 0.40 (0.326-70.0)	22.9, 17.2 (4.4-153.0)
No. glasses day		4.7, 4 (1-16)	
**Cooking water source**			
Tap	155 (80%)	29.9, 18.1 (0.326-236.0)*	42.9, 28.1 (4.3-319.7)*
Bottled water	38 (20%)	7.0, 0.653, (0.326-37.1)	18.5, 16.2 (4.4-63.5)
**Bathing water source**			
Tap	197 (100%)	N/A	N/A
Bottled water	0	N/A	N/A
**Seafood consumption**			
Consumed	43 (22%)	N/A	35.0, 22.0 (4.3-261.4)
Not consumed	155 (78%)	N/A	47.1, 28.1 (6.0-319.7)
**Age**			
<25	123 (61.5%)	27.0, 14.3 (0.326-236.0)	38.1, 22.6 (4.3-319.7)
25-30	50 (25%)	16.4, 3.4 (0.326-131.9)	34.9, 22.7 (4.4-170.2)
>30	27 (13.5%)	29.0, 12.8 (0.326-189.8)	39.5, 24.3 (7.3-224.3)
**Education**			
<High school	50 (25.2%)	31.3, 20.5 (0.326-226.2)*	47.7, 31.6 (7.8-224.3)*
High school	95 (47.7%)	24.6, 12.8 (0.326-212.1)	37.2, 24.6 (4.3-319.7)
College	41 (20.6%)	20.7, 8.80 (0.326-236.0)	32.1, 18.8 (7.4-153.0)
>College	13 (6.5%)	13.3, 0.635 (0.326-60.0)	19.4, 15.6 (7.3-53.4)
**Residence**			
Rural	61 (30.5%)	15.2, 13.2 (0.326-68.7)	30.8, 24.6 (4.3-153.0)
Urban	137 (68.5%)	32.8, 12.8 (0.326-236.0)	43.4, 22.6 (4.4-319.7)

[a]Differences in *n* based on missing demographic data.

*Significant difference in means ($p<0.05$) between subcategories.

Table S2. Multivariable regression analyses between exposure and metabolism indicators (DW-iAs, SG-Adjusted: U-tAs, U-iAs, U-MMAs, U-DMAs, %iAs, %MMAs, and %DMAs) and birth outcomes/measures.

**Characteristic**	**DW-iAs β (*p*-value) [CI]**	**U-tAs β (*p*-value) [CI]**	**U-iAs β (*p*-value) [CI]**	**U-MMAs β (*p*-value) [CI]**	**U-DMAs β (*p*-value) [CI]**	**%iAs β (*p*-value) [CI]**	**%MMAs β (*p*-value) [CI]**	**%DMAs β (*p*-value) [CI]**
**Gestational age (wks)**								
Unadjusted								
β (*p*-value)	-0.0025 (0.2280)	-0.0029 (0.1499)	-0.056 (0.0949)	-0.054 (0.0741)	-0.0030 (0.1749)	0.0032 (0.8851)	-0.036 (0.2061)	0.0078 (0.5778)
[CI]	[-0.0065, 0.0061]	[-0.0068, 0.0010]	[-0.12, 0.0097]	[-0.11, 0.0052]	[-0.0074, 0.0013]	[-0.040, 0.047]	[-0.093, 0.0200]	[-0.020, 0.035]
Model I[a]								
β (*p*-value)	-0.0032 (0.1233)	-0.0036 (0.0724)	-0.069 (0.0367)*	-0.067 (0.0233)*	-0.0037 (0.0913)	-0.0006 (0.9792)	-0.043 (0.1282)	0.011 (0.4423)
[CI]	[-0.0072, 0.0009]	[-0.0075, 0.003]	[-0.13, -0.0043]	[-0.13, -0.0092]	[-0.0081, 0.0006]	[-0.043, 0.042]	[-0.098, 0.012]	[-0.017, 0.38]
Model III[b]								
β (*p*-value)	-0.0040 (0.1177)	-0.0067 (0.0830)	-0.090 (0.0137)*	-0.088 (0.0103)*	-0.0075 (0.0980)	0.0007 (0.9775)	-0.0393 (0.2178)	0.0087 (0.5689)
[CI]	[-0.0091,0.0010)	[-0.0117,0.0017]	[-0.16, -0.019]	[-0.15, -0.021)	[-0.013, 0.0018]	[-0.045, 0.046]	[-0.10, 0.023]	[-0.021, 0.039]
**Birth weight (g)**								
Unadjusted								
β (*p*-value)	0.066 (0.93)	-0.35 (0.6537)	-18.1 (0.1616)	-18.6 (0.1098)	-0.25 (0.7730)	-7.9 (0.3547)	-23.8 (0.0303)	9.3 (0.0852)
[CI]	[-1.5, 1.6]	[-1.9, 1.2]	[-43.4,7.2]	[-41.3, 4.2]	[-1.9, 1.4]	[-24.6, 8.8]	[-45.4, -2.3]	[-1.3, 19.9]
Model I[a]								
β (*p*-value)	-0.1 (0.8956)	-0.58 (0.4489)	-21.7 (0.0901)	-24.4 (0.0325)*	-0.49 (0.5674)	-6.4 (0.4424)	-24.5 (0.0226)*	8.9 (0.0928)
[CI]	[-1.7, 1.4]	[-2.1, 0.93]	[-46.8, 3.4]	[-46.8, -2.0]	[-2.2, 1.2]	[-22.9, 10.0]	[-45.6, -3.4]	[-1.5, 19.3]
Model II[c]								
β (*p*-value)	0.19 (0.8031)	-0.0260 (0.9721)	-10.3 (0.4094)	-14.9 (0.1802)	0.038 (0.9642)	-6.1 (0.4453)	-18.1 (0.0848)	7.7 (0.1307)
[CI]	[-1.3, -1.7]	[-1.5, 1.4]	[-34.8, 14.2]	[-36.7, 6.9]	[-1.6, 1.7]	[-21.6, 9.5]	[-38.7, 2.5]	[-2.3, 17.8]
Model III[b]								
β (*p*-value)	-0.25 (0.8025)	-0.84 (0.3994)	-19.5 (0.1704)	-23.7 (0.0731)	-0.79 (0.4859)	-4.8 (0.5913)	-23.1 (0.0569)	7.4 (0.2023)
[CI]	[-2.20, 1.70]	[-2.79,1.11]	[-47.3, 8.4]	[-49.7, 2.2]	[-3.0, 1.4]	[-22.3, 12.7]	[-46.8, 0.68]	[-4.0, 18.8]
**Newborn length (cm)**								
Unadjusted								
β (*p*-value)	-0.0051 (0.2659)	-0.0053 (0.2270)	-0.16 (0.0257)*	-0.098 (0.1399)	-0.0054 (0.2747)	-0.059 (0.2260)	-0.071 (0.2631)	-0.0054 (0.2747)
[CI]	[-0.014, 0.0039]	[-0.014, 0.0033]	[-0.31, -0.020]	[-0.23, 0.032]	[-0.015, 0.0043]	[-0.15, 0.036]	[-0.19, 0.053]	[-0.015, 0.0043]
Model I[a]								
β (*p*-value)	-0.0051 (0.2629)	-0.0055 (0.2204)	-0.16 (0.0265)*	-0.11 (0.0898)	-0.0054 (0.2732)	-0.054 (0.2642)	-0.082 (0.1908)	0.041 (0.1796)
[CI]	[-0.014, 0.0038]	[-0.014, 0.0033]	[-0.31, 0.019]	[-0.24, 0.018]	[-0.015, 0.0043]	[-0.15, 0.041]	[-0.21, 0.041]	[-0.020 ,0.10]

Characteristic	DW-iAs β (*p*-value) [CI]	U-tAs β (*p*-value) [CI]	U-iAs β (*p*-value) [CI]	U-MMAs β (*p*-value) [CI]	U-DMAs β (*p*-value) [CI]	%iAs β (*p*-value) [CI]	%MMAs β (*p*-value) [CI]	%DMAs β (*p*-value) [CI]
Model II[c]								
β (*p*-value)	-0.0033 (0.4455)	-0.0019 (0.6581)	-0.091 (0.2033)	-0.049 (0.4413)	-0.0017 (0.7204)	-0.052 (0.2539)	-0.050 (0.4046)	0.032 (0.2686)
[CI]	[-0.018, 0.0052]	[-0.010, 0.0064]	[-0.23, 0.049]	[-0.17, 0.076]	[-0.011, 0.0075]	[-0.14, 0.037]	[-0.17, 0.067]	[-0.025, 0.089]
Model III[b]								
β (*p*-value)	-0.0034 (0.5162)	-0.0076 (0.1467)	-0.1745 (0.1890)	-0.1427 (0.4120)	-0.0077 (0.1973)	-0.0438 (0.3535)	-0.0863 (0.1815)	0.0390 (0.2048)
[CI]	[-0.014, 0.0069]	[-0.018, 0.0027]	[-0.32, 0.029]	[-0.2796, 0.0057]	[-0.019, 0.0040]	[-0.14, 0.049]	[-0.21, 0.040]	[-0.021, 0.099]
**Head circumference (cm)**								
Unadjusted								
β (*p*-value)	-0.0020 (0.4479)	-0.0017 (0.5277)	-0.054 (0.2397)	-0.052 (0.1876)	-0.0015 (0.5976)	0.0051 (0.8572)	-0.029 (0.4336)	0.0044 (0.8062)
[CI]	[-0.0072, 0.0032]	[-0.0069, 0.0035]	[-0.14, 0.036]	[-0.13, 0.026]	[-0.0073, 0.0042]	[-0.050, 0.060]	[-0.10, 0.0432]	[-0.031, 0.040]
Model I[a]								
β (*p*-value)	-0.0026 (0.3329)	-0.0022 (0.3959)	-0.061 (0.1864)	-0.067 (0.0907)	-0.0021 (0.4646)	0.01 (0.7129)	-0.03 (0.4129)	0.0025 (0.8892)
[CI]	[-0.0077, 0.0026]	[-0.0074, 0.0029]	[-0.15, 0.030]	[-0.14, 0.011]	[-0.0079, 0.0036]	[-0.044, 0.065]	[-0.10, 0.041]	[-0.032, 0.037]
Model II[c]								
β (*p*-value)	-0.0021 (0.4232)	-0.0019 (0.4590)	-0.058 (0.2047)	-0.060 (0.1228)	-0.0018 (0.5315)	0.0090 (0.7446)	-0.023 (0.5308)	0.0012 (0.9438)
[CI]	[-0.0073, 0.0030]	[-0.0071, 0.0032]	[-0.15, 0.032]	[-0.14, 0.0163]	[-0.0075, 0.0039]	[-0.045, 0.063]	[-0.094, 0.048]	[-0.033, 0.036]
Model III[b]								
β (*p*-value)	-0.0016 (0.6331)	-0.0004 (0.9154)	-0.0418 (0.4177)	-0.0344 (0.4583)	.000001 (0.9978)	0.099 (0.7388)	-0.0122 (0.7642)	-0.0034 (0.8588)
[CI]	[-0.0081, 0.0050]	[-0.0072, 0.0065]	[-0.14, 0.059]	[-0.13, 0.056]	[-0.0077, 0.0077]	[-0.048, 0.068]	[-0.092, 0.068]	[-0.041, 0.035]
**Placental weight (g)**								
Unadjusted								
β (*p*-value)	0.15 (0.5105)	0.43 (0.1529)	0.99 (0.8023)	-0.18 (0.9571)	0.39 (0.1164)	-3.4 (0.1618)	-9.3 (0.0027)*	3.5 (0.0232)*
[CI]	[-0.30, 0.60]	[-0.12, 0.77)	[-6.8, 8.8]	[-6.9, 6.6]	[-0.098, 0.89]	[-8.1, 1.4)	[-15.4, -3.2]	[0.47, 6.5]
Model I[a]								
β (*p*-value)	0.12 (0.5892)	0.31 (0.1736)	0.73 (0.8544)	-1.3 (0.7101)	0.38 (0.128)	-3.2 (0.1889)	-9.8 (0.0015)*	3.5 (0.021)*
[CI]	[-0.32, 0.57]	[-0.14, 0.76]	[-7.1, 8.5]	(-8.0, 5.5)	[-0.11, 0.88]	[-7.9, 1.6]	[-15.8, -3.8]	[0.53, 6.5]
Model II[c]								
β (*p*-value)	0.12 (0.5888)	0.31 (0.1790)	0.53 (0.8936)	-1.3 (0.7040)	0.38 (0.1319)	-3.2 (0.1823)	-9.7 (0.0017)*	3.5 (0.0223)*
[CI]	[-0.33, 0.57]	(-0.14, 0.75)	[-7.3, 8.3]	[-8.0, 5.4]	[-0.11, 0.87]	[-7.9, 1.5]	[-15.7, -3.7]	[0.49, 6.4]
Model III[b]								
β (*p*-value)	0.13 (0.6533)	0.38 (0.1952)	0.68 (0.5355)	-1.4 (0.7111)	0.47 (0.1478)	-1.5 (0.5501)	-9.5 (0.0045)*	2.6 (0.1066)
[CI]	[-0.42, 0.68]	[-0.19, 0.95]	[-5.8, 11.2]	[-9.08, 6.19]	[-0.17, 1.1]	[-6.4, 3.4]	[-16.0, -2.9]	[-0.56, 5.8]

Characteristic	DW-iAs<br>β (*p*-value)<br>[CI]	U-tAs<br>β (*p*-value)<br>[CI]	U-iAs<br>β (*p*-value)<br>[CI]	U-MMAs<br>β (*p*-value)<br>[CI]	U-DMAs<br>β (*p*-value)<br>[CI]	%iAs<br>β (*p*-value)<br>[CI]	%MMAs<br>β (*p*-value)<br>[CI]	%DMAs<br>β (*p*-value)<br>[CI]
**APGAR score**								
Unadjusted								
β (*p*-value)	0 (0.9812)	0 (0.9013)	-0.011 (0.7989)	-0.00010 (0.9704)	0 (0.8758)	0 (0.9948)	0.0008 (0.8186)	-0.0002 (0.9133)
[CI]	[-0.005, 0.005]	[-0.0005,0.0004]	[-0.0095, 0.0073]	[-0.0074, 0.0072]	[-0.0005, 0.0006]	[-0.0052, 0.0051]	[-0.0059, 0.0075)	[-0.0035, 0.0031]
Model I[a]								
β (*p*-value)	0 (0.9873)	0 (0.8519)	-0.0013 (0.7689)	0.0004 (0.9164)	0.0001 (0.8274)	-0.0002 (0.9298)	0.011 (0.7399)	-0.0001 (0.9372)
[CI]	[-0.0005, 0.0005]	[-0.0004, 0.0005]	[-0.0097,0.0072]	[-0.0069, 0.0070]	[-0.0005, 0.0006]	[-0.0054, 0.0049]	[-0.0056, 0.078]	[-0.0034, 0.003]
Model II[c]								
β (*p*-value)	0 (0.9806)	0 (0.8825)	-0.0015 (0.7263)	0.0003 (0.9432)	0 (0.8564)	-0.0003 (0.9227)	0.0003 (0.9432)	-0.0001 (0.9282)
[CI]	[-0.005, 0.005]	[-0.0004,0.0005]	[-0.010, 0.0070]	[-0.0070, 0.0076]	[-0.0005, 0.0006]	[-0.0054, 0.0049]	[-0.0070, 0.0076]	[-0.0034, 0.0031]
Model III[b]								
β (*p*-value)	0 (0.9889)	0.0001 (0.8370)	-0.0018 (0.7364)	0.0005 (0.9227)	0.0001 (0.8024)	-0.0004 (0.9086)	0.0012 (0.7707)	-0.0001 (0.9725)
[CI]	[-0.0007, 0.0007]	[-0.0006, 0.0008]	[-0.012, 0.0087]	[-0.0090, 0.0099]	[-0.0007, 0.0009]	[-0.0064, 0.0057]	[-0.0071, 0.0095]	[-0.0040, 0.0039]

*Association ($p<0.05$) between birth outcomes and arsenic exposure indicators.

[a]Model I was adjusted for the following covariates: maternal age, education, smoking, and alcohol consumption. [b]Model III sensitivity analysis analyzed using non-seafood consumers (n=155), adjusted for the following covariates: maternal age, education, smoking, and alcohol consumption.

[c]Model II was adjusted for the following covariates: gestational age, maternal age, education, smoking, and alcohol consumption.

Supplemental Material, Figure S1

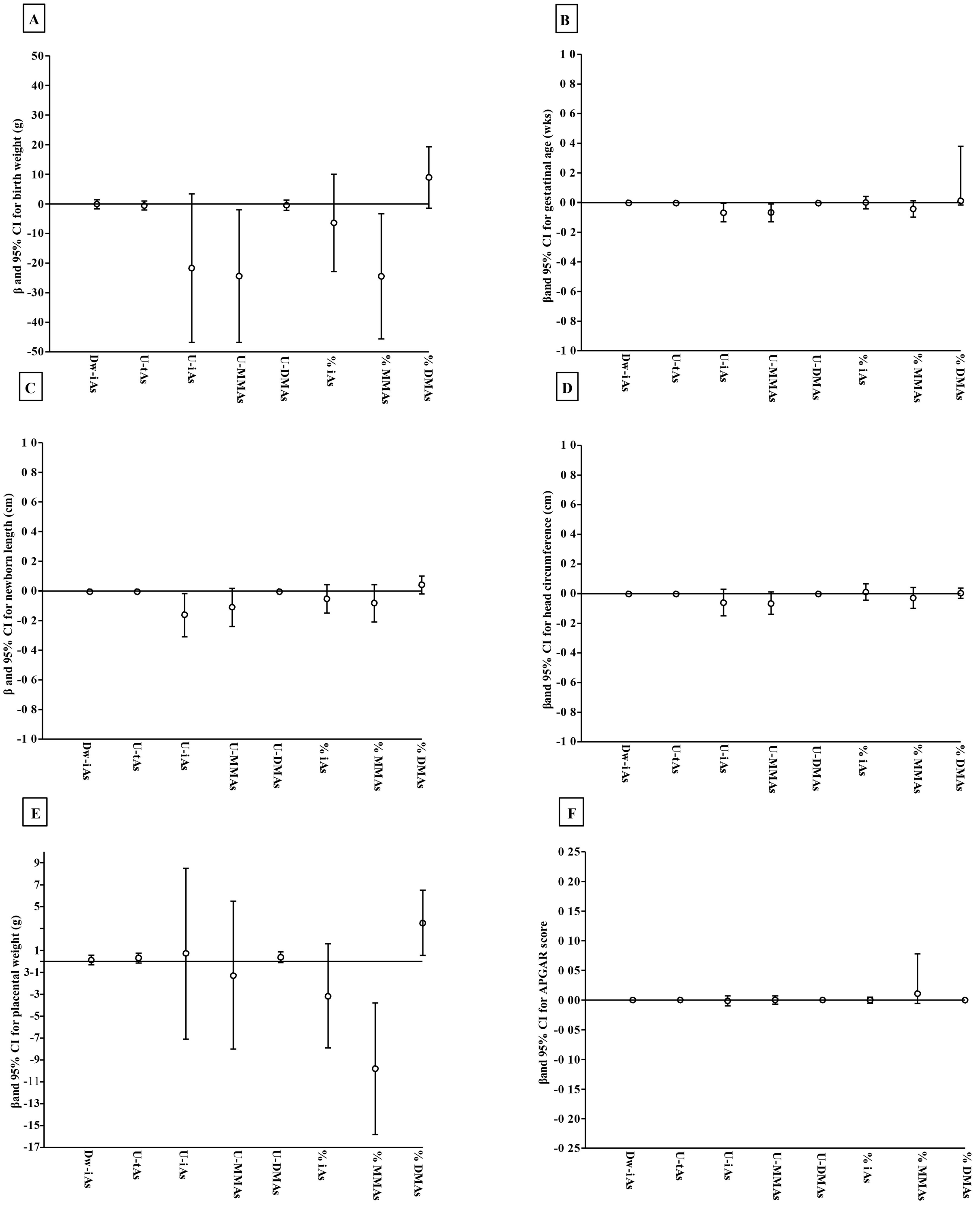

Figure S1. Multivariable regression analyses relating maternal iAs exposure and metabolism indicators and birth outcomes/measures. (A) birth weight, (B) gestational age, (C) newborn length, (D) head circumference, (E) placental weight, and (F) APGAR score. β estimates and confidence intervals are displayed.
